# Scientific American Special Navy Supplement

**Published:** 1898-05

**Authors:** 


### Scientific American Special Navy Supplement. Munn & Co., 361 Broadway, New York. Price 25 cents.

The lack of trustworthy information concerning our navy has induced the editors of the *Scientific American* to publish a Special Navy Supplement, which is certainly unique amongst the many magazines constituting our current periodical literature. Its handsome illustrations, its simple descriptions, enable one almost at a glance to comprehend the essential features in the construction and manipulation of our ships. In this admirable publication will be found our battleships "Indiana" and "Massachusetts," with their ponderous guns and powerful engines; the "Columbia" and "Minneapolis," destroyers of commerce; the monitors "Amphitrite" and "Miantonomah," illustrated by excellent sectional views, showing the construction and manipulation of their huge turrets and guns; the swift torpedo boats, "Porter" and "Bailey;" the "Vesuvius," with her three dynamite guns, and "Katahdin," with her formidable ram—both of them types of vessels found in no other navy of the world. The "Holland" submarine boat is also represented. To assist the reader in ascertaining the exact extent of Spanish possessions in the West Indies, an accurate, colored map of Cuba accompanies the paper. Says our own Captain Mahan: "With persons of average decision of character, and of average openness of mind, the wider the attention paid to the contemporaneous development of naval material under the advances of science, the more doubtful and ill-defined inclines to become the mental appreciation of existing conditions." It is this very perturbation of mind, this lack of clearness of thought regarding our warships, that a publication of this nature is well calculated to remove. Every effort has been made to treat the subject in a manner to be easily understood by those unversed in naval affairs, and in our judgment they have well accomplished the task. The illustrations number about ninety, and show the vessels grouped in classes, such as battleships, cruisers, monitors, gunboats, torpedo boats, special classes. Numerous pictures also illustrate, in great detail, guns, gun turrets, torpedoes, and many external and internal views of other parts of our warships. The whole forms a magnificent work of permanent and ready reference. It is sold for 25 cents by all newsdealers and by the publishers, Messrs. Munn & Co., 361 Broadway, New York.

